# Maternal grand multiparity and intention to use modern contraceptives in Nigeria

**DOI:** 10.1186/s12889-018-6130-1

**Published:** 2018-10-29

**Authors:** Bola Lukman Solanke, Olufunmilola Olufunmilayo Banjo, Bosede Odunola Oyinloye, Soladoye Sunday Asa

**Affiliations:** Department of Demography and Social Statistics, Obafemi Awolowo University, Ile-Ife, Nigeria

**Keywords:** Parity, Multiparity, Grand multiparity, Contraceptive use, Reproductive health, Nigeria

## Abstract

**Background:**

Grand multiparity and low contraceptive prevalence are dominant among Nigerian women. These elevate the risk of unintended pregnancies, high-risk fertility and adverse maternal and child health outcomes among women in the country, particularly grand multiparous women. Studies have examined predictors of intention to use modern contraceptives among women of reproductive age. However, these studies did not ascertain the extent to which grand multiparity is associated with intention to use modern contraceptives. This study examined association between grand multiparity and intention to use modern contraceptives in Nigeria.

**Methods:**

The study pooled data from 2003 to 2013 Nigeria Demographic and Health Surveys. The weighted sample size analysed was 34,302 women. The outcome variable was intention to use contraceptive. The main explanatory variable was parity with specific attention to grand multiparity. Unadjusted multinomial logistic regression coefficients were used to examine association between specific explanatory or control variables and intention to use contraceptives while the adjusted multinomial logistic regression was applied to further examine associated factors of intention to use contraceptives relative to being uncertain about future contraceptive use. Four multinomial logistic regression models were fitted using Stata 14.

**Results:**

More than half of respondents do not intend to use contraceptives, while less than one-fifth of respondents intend to use contraceptives in the future. Across the four fitted models, the relative risks of intention to use compared with being uncertain about future contraceptive use were significantly lower among grand multiparous women. Results further revealed pregnancy termination, fertility planning status, exposure to mass media family planning messages, knowledge of modern contraceptives, ideal family size, remarriage, household power relations, and maternal education as other key factors influencing expected risk of intention to use contraceptives relative to being uncertain about future contraceptive use.

**Conclusion:**

Maternal grand multiparity is significantly associated with intention to use contraceptives among women in Nigeria. The development of a specific population and health programme to target grand multiparous women is imperative in the country. Such programme could be integrated into existing national family planning programme through specific contraceptive education, counselling and information for high parous women.

## Background

Parity is the number of live births borne by a woman, and may be categorised into primiparity (one live birth), multiparity (more than one but less than five live births), and grand multiparity which is also referred to as high parity (five or more live births) [1–3]. Parity has been identified as an independent factor that elevates the risks of childbearing particularly the risks associated with pregnancy and childbirth [4–10]. This however is with regard to specific categories of parity. The grand multiparous condition has attracted more research attention due to increasing evidence of higher likelihood of adverse maternal and newborn health outcomes among grand multiparous women. This attention first emanated from the works of Bethel Solomon who used the term “The Dangerous Multipara” to correct erroneous impression that previous experience of childbirth eases the burden of child delivery [11, 12]. Subsequently, several obstetric studies reviewed cases of child deliveries in hospitals for the purpose of determining whether grand multiparity was truly an obstetric risk. In this regard, a large number of studies across the world have provided empirical evidence that grand multiparity indeed remains an obstetric risk [13–20].

Though few studies [21] have argued that grand multiparity may not be discouraged if women are provided with adequate perinatal care, the health risks of grand multiparity include but not limited to incidence of anaemia in pregnancy [22], type 2 Diabetes [23], severe mental disorders [24], perinatal death [25], and several prenatal risks [26]. Incidentally, grand multiparity is more prevalent in developing countries where health delivery systems are grossly inadequate to cope with rising demand for maternal and child health problems [27–30]. In Nigeria, having large number of children is highly valued across communities. Motherhood to a great extent determines how a woman is treated or respected in many Nigerian communities [31]. In several cases, women facing fertility challenges also face increased likelihood of stigmatisation and intimate partner violence [32–34]. Indeed, in many Nigerian communities, childbearing is widely perceived as the sole work of women in the communities [35], and voluntary or involuntary childlessness are not accepted in virtually every part of Nigeria [32, 36]. Several fertility studies in the country have thus documented the dominance of grand multiparity among Nigerian women [37, 38] with evidence that many grand multiparous women seek medical treatment for delays experienced in the process of having more children [39]. Also, a number of obstetric studies in the country concluded that grand multiparity remains a public health challenge in Nigeria [40–44].

Against this backdrop, studies have observed that the prevalence of grand multiparity present key challenges for scaling up family planning programmes to improve men and women’s contraceptive behaviour with the purpose of reducing high and unintended pregnancies and high-risk fertility among women particularly grand multiparous women [3, 29]. This is based on the understanding that many women in developing countries including grand multiparous women do not use modern contraceptives for various reasons [45–51]. It also implies that many grand multiparous women still face the risk of unintended fertility which may not only further aggravate their reproductive health and well-being, but also sustain current high birth rates in developing countries including Nigeria.

However, in spite of these implications, studies have rarely examined the extent to which grand multiparity is associated with intention to use modern contraceptives among women. Though studies have examined predictors of intention to use modern contraceptives among women of reproductive age [52–54], these studies did not ascertain the extent to which grand multiparity is associated with intention to use modern contraceptives. This has not only limited adequate assessment of future use of contraceptives among grand multiparous women. It also undermines the knowledge of the extent to which grand multiparity may influence future contraceptive use. This study addressed these limitations by raising the question: to what extent is grand multiparity associated with intention to use modern contraceptives? The objective of the study was to examine association between grand multiparity and intention to use modern contraceptives. This was with the view to drawing attention to the implications of grand multiparity for future contraceptive behaviour and women’s reproductive health and general well-being in the march towards attainment of the Sustainable Development Goals (SDGs) in Nigeria. Also, this study provides additional information that specific family planning strategy could be developed to target grand multiparous women in the country. This is important for repositioning the existing 2004 National Population Policy for Sustainable Development, which is yet to make appreciable impact on quality of life in the country [55–57].

The Theory of Planned Behaviour (TPB) which is an extension of the Theory of Reasoned Action (TRA) provided the theoretical focus for this study. The TPB asserts that the proximate determinant of behaviour is behavioural intentions, which depends on attitude toward the behaviour, social normative perception and perceived behaviour control [58–60]. Though, there are arguments against the use of the TPB for modelling human fertility [61], the TPB have been widely verified to have high capacity for predicting human behavioural intention [62, 63]. There are three key constructs of the TPB [63]. The first is behaviour belief (perceived positive or negative consequences of a specific behaviour) that results in specific attitudes upon individual evaluation of performing the behaviour (behaviour attitudes). The second is normative belief (expectation and behaviours of important element in the household or society) that results in the degree to which an individual wants to agree or disagree with the expectation of the important elements (subjective norm). The third is control belief (external factors that may hinder or enhance the performance of the behaviour) which results in the assessment of how easy or difficult it is to perform the behaviour (perceived behaviour control).

Behaviour belief in this study was proxy by child mortality, type of marriage and ideal family size. These factors may directly affect parity or indirectly affect parity by encouraging or discouraging intention to use contraceptives through reproductive attitudes. For instance, most women who have experienced child mortality usually have positive attitudes towards replacing the dead child and are likely to want to replace the dead child [3]. Also, co-wives competition within polygynous household may affect intention to use contraceptives by impacting parity [64] through reproductive attitudes that discourage intention to use contraceptives. Given that contraceptive use is low among Nigerian women, desire of the individual woman not to have a pregnancy termination or a repeat pregnancy termination will impact intention to use contraceptives. Studies have already shown that contraceptive use increases when post-abortion family planning services are provided following pregnancy termination [65, 66].

Normative belief was proxy in the study by remarriage and fertility desire. These factors will influence the extent to which women meets partner or societal expectation for additional children. For instance, regardless of previous number of children, remarried women in most parts of Nigeria are expected to raise additional children in the new marriage for the purpose of optimal marital satisfaction [67]. This may affect their parity with effect on intention to use contraceptives. Also, evidence has shown that men have higher fertility desire than women in Nigeria [68, 69], and given male dominance of reproductive decisions in Nigerian households [70], many women do not have power to plan their fertility. The occurrence of a mistimed or unwanted pregnancy among such women is more likely to increase their parity which may create need to evaluate future use of contraceptives. Control belief in the study was proxy by household power relations and maternal education. These factors represent the extent to which women may independently access contraceptive information or services without or with little male partner involvement.

## Methods

### Data source, pooling and sample size

The study pooled data from Individual Recode (Women data) of 2003, 2008, and 2013 Nigeria Demographic and Health Surveys (NDHSs). The essence of pooling data from the 2003–2013 NDHSs is to analyse a sufficiently large sample to improve statistical precision and understanding of the relationship between parity and intention to use contraceptives. Pooling data from nationally representative samples is particularly suitable because the samples have been collected using similar design**s** with most of the variables being assigned the same names and codes. Increasing number of studies [71–74] has adopted the data pooling approach particularly in the analyses of data from the DHS Programme. The National Population Commission (NPC) implemented the surveys in Nigeria with both technical and financial assistance of ICF International. The surveys were nationally representative. In each of the surveys, samples were selected through multi-stage cluster designs. The detail designs employed in the surveys have been widely published [69, 75, 76]. The pooled data covered a total of 79,953 women aged 15–49 years. However, the current study did not analyse all the women. All nulliparous women (that is women who have not had a live birth) were excluded because the unit of analysis in the study are women who have had at least a live birth. The eligibility criteria in the study are having had at least one live birth, being sexually active in the last one month, not being declared infecund, not currently using a modern contraceptive method, and being currently married. The weighted sample size analysed was 34,302 women.

### Research variables

The outcome variable in the study was intention to use modern contraceptives. This variable measures the extent to which non-users of modern contraceptives plan to use any modern method in the future [69]. This information was sourced from currently married women who were not using a modern method. The variable has three categories, namely, (1) use later, (2) uncertain, and (3) does not intend to use. However, analyses in the study focused on ‘use later’ category with the ‘uncertain’ category as the base category. The reason for this focus is the importance of the ‘use later’ category since women in this category are those who are more likely to demand contraceptive method in the future. The main explanatory variable was parity with specific attention to grand multiparity. Other explanatory variables are based on the theoretical constructs of the TPB. Descriptions of these variables are presented in Table 1. Five individual characteristics, namely, place of residence, maternal age, religion, age at first birth, and geographic region are selected for statistical control.

**Table 1:** Variable Identification and Description

S/No.	Name of Variable	Description	Categories
Outcome Variable			
1.	Intention to use contraceptive	Future plan for modern contraceptive	1. Use later 2. Uncertain 3. Does not intend
Main Explanatory Variable			
2.	Parity	Number of children borne by respondents	1. Primiparity 2. Multiparity 3. Grand Multiparity
Background Characteristics			
3.	Place of residence	Type of community where respondents resides	1. Urban 2. Rural
4.	Maternal age	Current age group of respondents	1. 15–24 years 2. 25–34 years 3. 35 years or older
5.	Age at first birth	Respondents age at commencement of motherhood	1. 15–19 years 2. 20–24 years 3. 25 years or older
6.	Religion	Religious affiliation of respondents	1. Christianity 2. Islam 3. Traditional/others
7.	Geographical region	Geographic location of respondents	1. Northern region 2. Southern region
Behaviour Belief			
8.	Child mortality experience	Respondents experience of death of a child	1. Ever experienced 2. Never experienced
9.	Type of marriage	Number of wives of respondents husband	1. Monogamy 2. Polygyny
10.	Ideal family size	The number of children a woman want to have if she has the means of choosing the number	1. 1 or less 2. 2 3. 3+ 4. 4 5. 5 or more
Normative Belief			
11.	Remarriage	Number of marriages	1. Once 2. More than once
12.	Partner fertility desire	The fertility desire of the male partner compared with that of the woman	1. Same 2. Partner wants more 3. Partner wants fewer
Control Beliefs			
13.	Household power relations	Type of dominance in household decision-making	1. Male dominated 2. Not male dominated
14.	Maternal education	Respondents educational attainment	1. None 2. Primary 3. Secondary 4. Higher
Behaviour attitudes			
15.	Previous contraceptive status	Whether a woman has ever or never used a method	1. Ever used 2. Never used
16.	Pregnancy termination	Whether respondents has ever terminated a pregnancy irrespective of cause	1. Ever experienced 2. Never experienced
Subjective Norm			
17.	Fertility planning status	Planning status of the most recent birth	1. Wanted 2. Mistimed 3. Unwanted
Perceived Behaviour Control			
18.	Family planning media message exposure	The main source of mass media family planning message	1. None 2. Radio 3. Television 4. Newspaper
19.	Knowledge of contraceptive	Knowledge of any contraceptive method	1. No method 2. Traditional method 3. Modern method

### Data analysis

Statistical analyses were carried out at three levels in the study. At the univariate level, frequency distribution, percentages and charts were used to described sample characteristics and intention to use contraceptives. At the bivariate level, unadjusted multinomial logistic regression coefficients were used to examine the association (positive or negative) between specific explanatory or control variable and intention to use contraceptive. At the multivariate level, the adjusted multinomial logistic regression was used to further examine association between the research variables. To be selected for inclusion in the multivariate analysis, a variable must have shown statistical significance with at least one outcome category at the bivariate level.

The multinomial logistic regression is most suitable for the study because the three categories of the outcome variable do not have natural ordering. If the categories are ordered, the ordered logistic regression will also be suitable for the study. However, the shortcoming of the ordered logistic regression is that it will not provide separate regression coefficients for each category of the outcome variable; hence the multinomial logistic regression was preferred. Four multinomial regression models were fitted in the study using the *mlogit command* of Stata version 14 [77]. Model 1 was based solely on parity while controlling for other explanatory variables. Model 2 included parity, measures of behaviour attitude, subjective norm and perceived behaviour control while controlling for the underlying determinants and the background characteristics. Model 3 included parity, and the underlying and proximate factors while controlling for the background characteristics. Model 4 was the full model and included all explanatory and control variables. The Relative Risk Ratio (RRR) was used to estimate the multinomial regression coefficients. The RRR measured change in the ‘use later’ category in relation to the ‘uncertain’ category due to change in the explanatory variable. The survey weights provided by the DHS were applied during the regression analysis. Prior to performing the multivariate analysis, a multi-collinearity check was carried out using the mean score of Variance Inflation Factor (VIF). This test was carried out by applying Stata command ‘vif uncentered’. The mean VIF score of 3.16 obtained confirm that there is no substantial multicollinearity among the explanatory variables [78]. Statistical significance in the study was set at 5%.

### Ethics statement

The surveys were approved by the National Health Research Ethics Committee with assigned number NHREC/01/01/2007 in the 2008 and 2013 surveys [69, 76]. Authorisation to download and analyse the datasets was obtained from MEASURE/DHS through on-line application. Analyses are not linked to any individual or household.

## Results

### Univariate analysis

**Fig. 1:**
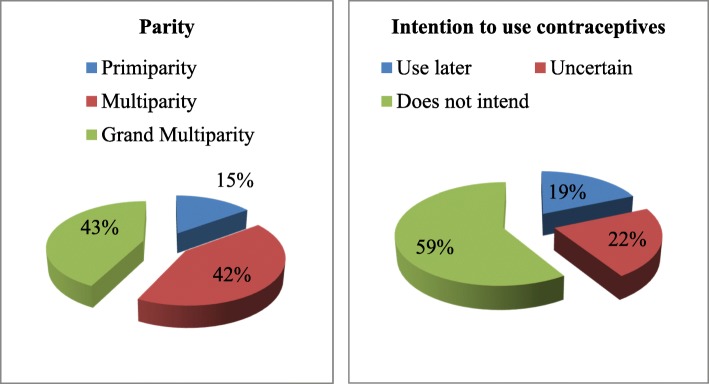
Percentage Distribution of Respondents by Parity and Intention to use contraceptives

Fig. 1 presents distribution of parity and intention to use contraceptives among respondents. Less than one-fifth of respondents were primiparous while more than one-third of respondents were either in the multiparous or grand multiparous categories though with slightly more proportion of respondents in the grand multiparous category. More than half of respondents do not intend to use contraceptives in the future; nearly one-fifth intend to use contraceptives in the future, while slightly more than one-fifth was unsure of use in the future. Table 2 presents the socio-demographic characteristics of respondents. More than two-thirds of respondents reside in rural areas while less than one-third of them reside in urban areas of the country. The majority of respondents are currently in the age interval of 25–34 years. However, almost two-fifths of respondents are in the advanced reproductive age 35 years or older. The majority of respondents were in the age interval 15–19 years at their first birth. Muslim women particularly from the Northern region were dominant in the sample. Though, more than half of respondents had never experienced child mortality, a substantial proportion of respondents had ever experienced child mortality. Monogamy was the dominant type of marriage among respondents. The majority of respondents desired to have five or more children, and had been married only once.

**Table 2:** Socio-Demographic Characteristics of Respondents, 2003–2013 NDHS, Nigeria

Characteristic	Number of Women (*n* = 34,302)	Percentage
Place of Residence		
Urban	10,446	30.5
Rural	23,836	69.5
Maternal age		
15–24 years	7220	21.1
25–34 years	14,004	40.8
35 years or older	13,078	38.1
Age at first birth		
15–19 years	21,856	63.7
20–24 years	8577	25.0
25 years or older	3869	11.3
Religion		
Christianity	10,489	30.6
Islam	21,009	61.2
Traditional/others	2804	8.2
Geographic Region		
Northern	24,829	72.4
Southern	9473	27.6
Child Mortality		
Ever experienced	14,603	42.6
Never experienced	19,699	57.7
Type of Marriage		
Monogamy	21,566	62.9
Polygyny	12,736	37.1
Ideal Family Size		
1	406	1.2
2	248	0.7
3	858	2.5
4	4041	11.8
5+	28,749	83.8
Remarriage		
No	29,264	85.3
Yes	5038	14.7
Fertility Desire		
Both want same	10,472	30.5
Husband want more	13,294	39.8
Husband want fewer	1316	3.8
Uncertain	9220	26.9
Household Power Relations		
Not male dominated	8859	25.8
Male dominated	25,443	74.2
Maternal Education		
None	19,397	56.5
Primary	6280	18.3
Secondary	6766	19.7
Higher	1859	5.4
Previous Contraceptive Status		
Never used	31,596	92.1
Ever used	2706	7.9
Pregnancy Termination		
Never experienced	29,308	85.4
Ever experienced	4994	14.6
Planning Status of Last Birth		
Wanted	32,309	94.2
Mistimed	1293	3.8
Unwanted	700	2.0
Exposure to Mass Media Family Planning		
No exposure	22,805	66.5
Radio	6041	17.6
Television	3610	10.5
Newspaper	1846	5.4
Knowledge of Family Planning Method		
None	8615	25.1
Traditional	687	2.0
Modern	25,000	72.9

Source: Authors’ analysis based on 2003–2013 Nigeria Demographic and Health Survey

Fertility desire was higher among respondents’ partners compared with the surveyed women. In the majority of respondents’ households, power relations in terms of household decision-making were male dominated. Education was poor among respondents with almost three out of five women reporting no formal education, and less than one-fifth each reporting either primary or secondary education. Higher education was the least attained among respondents. The majority of respondents had never used contraceptive. More than one tenth of respondents had ever experienced a pregnancy termination, but the majority of respondents wanted their most recent live birth at the time the pregnancy occurred. Though, more than two-thirds of respondents know at least a modern contraceptive method, exposure to mass media family planning messages was poor among respondents with more than two-thirds of respondents reporting no exposure to mass media family planning messages.

### Bivariate analysis

Table 3 presents the association between the research variables. Parity was negatively associated with to intention to use contraceptives later with lowest intention to use contraceptives observed among grand multiparous women compared with other parous women Place of residence and intention to use contraceptives later are negatively associated (β = − 0.135; 95% CI: -0.204, − 0.065). Rural women compared with urban women (15.6% vs. 25.4%) had lower proportion of intention to use contraceptives later. The association between maternal age and intention to use contraceptives later was mixed. The association was positive among younger women (β = 0.160; 95% CI: 0.071–0.248) but negative (β = − 120; 95% CI: -0.215, − 0.025) among women in advanced reproductive age among whom the lowest proportion of intention to use contraceptives was observed. Age at first birth and intention to use contraceptives later were positively related. The proportion of women who intend to use contraceptives later increased as age at first birth increases. Moslem women compared with Christian women showed less intention to use contraceptives later (12.0% vs. 32.6%) indicating a negative association between religion and intention to use contraceptives later. Geographic region and intention to use contraceptives later are positively associated (β = 0.178; 95% CI: 0.110–0.246) with higher intention to use contraceptives observed among Southern women compared with Northern women (31.2% vs. 13.8%). The association between child mortality and intention to use contraceptives later was positive (β = 0.06; 95% CI: -0.044, 0.095). Women who had never experienced child mortality had higher intention to use contraceptives later compared with women who had ever experienced child mortality (21.0% vs. 15.4%).

**Table 3:** Percent intention to use contraceptive later by respondents’ characteristics and unadjusted multinomial logistic regression coefficients

Characteristic	Use Contraceptives Later			
	%	Coeff.	p	95% CI
Parity				
Primiparity ^ref^	23.3	–	–	–
Multiparity	20.7	−0.245	< 0.001	−0.341, − 0.148
Grand Multiparity	14.9	−0.306	< 0.001	− 0.407, − 0.205
Place of Residence				
Urban ^ref^	25.4	–	–	–
Rural	15.6	−0.135	< 0.001	−0.204, − 0.065
Maternal age (years)				
15–24 ^ref^	18.3	–	–	–
25–34	23.1	0.160	< 0.001	0.071–0.248
35+	14.0	−0.120	0.013	−0.215, − 0.025
Age at first birth (years)				
15–19 ^ref^	15.5	–	–	–
20–24	23.7	0.147	< 0.001	0.071–0.223
25+	24.7	0.075	0.143	−0.025-0.175
Religion				
Christianity ^ref^	32.6	–	–	–
Islam	12.0	−0.542	< 0.001	− 0.612,-0.472
Traditional/others	15.5	−0.069	0.365	−0.219-0.081
Geographical Region				
Northern ^ref^	13.8	–	–	–
Southern	31.2	0.178	< 0.001	0.110–0.246
Child Mortality				
Ever experienced ^ref^	15.4	–	–	–
Never experienced	21.0	0.026	0.473	−0.044, 0.095
Type of Marriage				
Monogamy ^ref^	21.4	–	–	–
Polygyny	13.9	−0.123	< 0.001	−0.196, − 0.049
Ideal Family Size				
1 ^ref^	7.9	–	–	–
2	27.4	1.046	< 0.001	0.543–1.545
3	38.5	1.374	< 0.001	0.973–1.774
4	34.8	1.231	< 0.001	0.856–1.606
5+	15.8	0.889	< 0.001	0.520–1.258
Remarriage				
No ^ref^	14.0	–	–	–
Yes	16.6	0.127	0.012	0.028–0.225
Fertility Desire				
Both want same ^ref^	25.6	–	–	–
Husband want more	14.1	−0.198	< 0.001	−0.281, −0.114
Husband want fewer	29.4	−0.097	0.231	−0.256-0.052
Uncertain	15.7	−0.768	< 0.001	−0.853, − 0.683
Household Power Relations				
Male dominated ^ref^	25.6	–	–	–
Not male dominated	16.2	−0.057	0.111	−0.128-0.013
Maternal Education				
None ^ref^	9.6	–	–	–
Primary	25.7	0.716	< 0.001	0.626–0.806
Secondary	33.9	0.726	< 0.001	0.643–0.810
Higher	33.7	0.530	< 0.001	0.404–0.656
Previous Contraceptive Status				
Never used ^ref^	17.3	–	–	–
Ever used	34.3	−0.269	< 0.001	−0.361, − 0.177
Pregnancy Termination				
Never experienced ^ref^	17.8	–	–	–
Ever experienced	23.3	0.294	< 0.001	0.201–0.387
Fertility Planning Status				
Wanted ^ref^	17.4	–	–	–
Mistimed	43.8	0.684	< 0.001	0.546–0.822
Unwanted	29.4	0.236	0.022	0.034–0.437
Family Planning Mass Media Messages				
No exposure ^ref^	14.0	–	–	–
Radio	21.4	0.455	< 0.001	0.362–0.548
Television	34.5	0.572	< 0.001	0.476–0.668
Newspaper	34.5	0.484	< 0.001	0.360–0.607
Knowledge of Family Planning Method				
No method ^ref^	4.4	–	–	–
Traditional	9.9	0.478	0.002	0.171–0.784
Modern	23.7	1.368	< 0.001	1.255–1.482

Type of marriage and intention to use contraceptives later are negatively associated (β = − 0.123; 95% CI: -0.196, − 0.049) with higher intention to use contraceptives later observed among monogamous women compared with polygynous women (21.4% vs. 13.9%). The association between ideal family size and intention to use contraceptives later was positive but the proportion of women who intend to use contraceptives later was inconsistent as ideal family size increases. Remarriage and intention to use contraceptives later are positively associated (β = 0.127; 95% CI: 0.028–0.225) with slightly higher intention to use contraceptives in the future among remarried women. The association between fertility desire and intention to use contraceptives later was negative. Intention to use contraceptives later was highest among women whose male partners wanted fewer children, but women whose male partners had desire for more children had the least intention to use contraceptives later. Household power relation was negatively associated with intention to use contraceptives later (β = − 0.057; 95% CI: -0.128, − 0.013). However, women who lived in households where power relations were male dominated had higher intention to use contraceptives later compared with those in households without male dominance of the power relations.

Intention to use contraceptives later substantially improves as maternal education improved, thus revealing a positive association between maternal education and intention to use contraceptives later. Previous contraceptive status was negatively related to intention to use contraceptives later. Higher prevalence of intention to use contraceptives later was observed among women who had ever used contraceptive compared to women who never used a method (23.3% vs. 17.3%). The relationship between pregnancy termination and intention to use contraceptives later was positive (β = 0.294; 95% CI: 0.201–0.387), and showed that women who had ever terminated a pregnancy were more willing to use contraceptives later. Fertility planning status relates positively with intention to use contraceptives. Higher level of intention to use contraceptives was observed among women whose last child was mistimed. Television and newspaper family planning messages were positively associated with higher intention to use contraceptives later. Likewise, knowledge of modern contraceptive method was associated with higher intention to use contraceptives later. These bivariate associations were further investigated at the multivariate level.

### Multivariate analysis

Results of the multivariate analysis are presented in Table 4. However, only results for intention to use contraceptive later are presented in the section in line with the study objective. In Model 1 based solely on parity, a change in parity from primiparity to grand multiparity was associated with a decrease in the relative risk of intention to use contraceptives later compared with being uncertain (RRR = 0.736; 95% CI: 0.666–0.814). With the inclusion of the proximate determinants in Model 2, and controlling for the underlying determinants, the expected intention to use contraceptives later relative to being uncertain was lower among grand multiparous women (RRR = 0.762; 95% CI: 0.687–0.844). In the model, the relative risk of intention to use contraceptives later relative to being uncertain was reduced by a factor of 0.552 among women who had ever used contraceptives (RRR = 0.552; 95% CI: 0.502–0.608). However, the relative risk of intention to use contraceptives later compared with being uncertain increased significantly among women who: ever had a pregnancy termination (RRR = 1.238; 95% CI: 1.126–1.361); had either a mistimed (RRR = 1.883; 95% CI: 1.635–2.167) or unwanted last child (RRR = 1.273; 95% CI: 1.036–1.563); had exposure to mass media family planning messages; and had knowledge of modern contraceptive method (RRR = 3.758; 95% CI: 3.340–4.229).

**Table 4:** Factors predicting intention to use contraceptives later relative to being uncertain about future contraceptive use

Characteristic predicting intention to use later	Model 1		Model 2		Model 3		Model 4	
	RRR	95% CI	RRR	95% CI	RRR	95% CI	RRR	95% CI
Main explanatory variable: Parity								
Primiparity ^ref^	–	–	–	–	–	–	–	
Multiparity	0.783*	0.710–0.863	0.788*	0.714–0.870	0.792*	0.716–0.877	0.790*	0.708–0.881
Grand Multiparity	0.736*	0.666–0.814	0.762*	0.687–0.844	0.796*	0.707–0.893	0.862**	0.745–0.997
Proximate variables: Previous Contraceptive Status								
Never used ^ref^			–	–	–	–	–	–
Ever used			0.552*	0.502–0.608	0.493*	0.446–0.546	0.520*	0.470–0.576
Pregnancy Termination								
Never experienced ^ref^			–	–	–	–	–	–
Ever experienced			1.238*	1.126–1.361	1.197*	1.087–1.317	1.205*	1.093–1.328
Fertility Planning Status								
Wanted ^ref^			–	–	–	–	–	–
Mistimed			1.883*	1.635–2.167	1.809*	1.568–2.086	1.721*	1.489–1.989
Unwanted			1.273**	1.036–1.563	1.206*	0.979–1.486	1.248**	1.012–1.540
Family Planning Mass Media Messages								
No exposure ^ref^			–	–	–	–	–	–
Radio			1.339*	1.217–1.473	1.267*	1.151–1.395	1.355*	1.229–1.494
Television			1.466*	1.325–1.621	1.261*	1.132–1.404	1.468*	1.313–1.642
Newspaper			1.337*	1.176–1.520	1.173**	1.018–1.352	1.321*	1.143–1.527
Knowledge of Family Planning Method								
No method ^ref^			–	–	–	–	–	–
Traditional			1.616**	1.189–2.197	1.636**	1.201–2.229	1.654**	1.213–2.256
Modern			3.758**	3.340–4.229	3.154*	2.793–3.563	3.168*	2.803–3.581
Underlying variables: Child Mortality								
Ever experienced ^ref^					–	–	–	–
Never experienced					0.888**	0.818–0.963	0.907**	0.836–0.984
Type of Marriage								
Monogamy ^ref^					–	–	–	–
Polygyny					1.012	0.932–1.098	1.048	0.964–1.139
Ideal Family Size								
1 ^ref^					–	–	–	–
2					1.446	0.860–2.430	1.454	0.862–2.452
3					1.636**	1.078–2.433	1.626**	1.068–2.474
4					1.446	0.979–2.135	1.493**	1.009–2.211
5+					1.308	0.894–1.912	1.316	0.899–1.927
Remarriage								
No ^ref^					–	–	–	–
Yes					1.225*	1.112–1.372	1.253*	1.127–1.393
Fertility Desire								
Both want same ^ref^					–	–	–	–
Husband want more					0.945	0.863–1.035	0.968	0.882–1.062
Husband want fewer					0.902	0.767–1.062	0.910	0.772–1.073
Uncertain					0.510*	0.466–0.558	0.510	0.465–0.559
Household Power Relations								
Male dominated ^ref^					–	–	–	–
Not male dominated					1.151*	1.065–1.243	1.148**	1.061–1.243
Maternal Education								
None ^ref^					–	–	–	–
Primary					1.708*	1.550–1.883	1.712*	1.540–1.903
Secondary					1.649*	1.395–1.720	1.578*	1.404–1.773
Higher					1.224*	1.044–1.435	1.269**	1.071–1.504
Background variables: Place of Residence								
Urban ^ref^							–	–
Rural							1.103**	1.018–1.195
Maternal age (years)								
15–24 ^ref^							–	–
25–34							1.109	0.994–1.238
35+							0.835**	0.726–0.960
Age at first birth (years)								
15–19 ^ref^							–	–
20–24							1.013	0.930–1.105
25+							0.916	0.870–1.040
Religion								
Christianity ^ref^							–	–
Islam							0.628*	0.568–0.694
Traditional/others							0.975	0.823–1.154
Geographical Region								
Northern ^ref^							–	–
Southern							0.624	0.566–0.687

Notes: ref. (reference category), *p < 0.001, **p < 0.05

In Model 3, the relative risk of intention to use contraceptives later compared with being uncertain reduced significantly among grand multiparous women (RRR = 0.796; 95% CI: 0.707–0.893) and multiparous women (RRR = 0.792; 95% CI: 0.716–0.877). In the model, the variables associated with significant reduction in the relative risk of intention to use contraceptives compared to being uncertain are previous contraceptive status, child mortality experience, and fertility desire. On the other hand, the variables associated with higher relative risk of intention to use contraceptives later compared with being uncertain are pregnancy termination, fertility planning status, exposure to mass media family planning messages, ideal family size, remarriage, household power relation, and maternal education. For instance, the expected intention to use contraceptives later compared to being uncertain was more than three times higher among women who had knowledge of modern contraceptive (RRR = 3.154; 95% CI: 2793–3.563). Likewise, the relative risk of intention to use contraceptives later compared with being uncertain was higher among remarried women (RRR = 1.235; 95% CI: 1.112–1.372).

In the full model, parity was negatively associated with intention to use contraceptives later. The expected risk of intention to use contraceptives later relative to being uncertain about future use significantly reduced among multiparous (RRR = 0.790; 95% CI: 0.708–0.881) and grand multiparous (RRR = 0.862; 95% CI: 0.745–0.997) women. With the exclusion of previous contraceptive status, all the proximate factors were associated with higher likelihood of intention to use contraceptives later. For instance, among women who had ever terminated a pregnancy compared with those who had never terminated pregnancy, the expected risk of intention to use contraceptives later relative to being uncertain about future use was higher by 20.5% (RRR = 1.205; 95% CI: 1.093–1.328). Similarly, among women exposed to newspaper family planning messages compared with non-exposed women, the relative risk of intention to use contraceptives later versus being uncertain about future use was higher by 32.1% (RRR = 1.321; 95% CI: 1.143–1.527).

With the exclusion of child mortality, all the underlying factors were associated with higher likelihood of intention to use contraceptives later. For instance, the relative risk of intention to use contraceptives later compared with being uncertain was 25.3% higher among women who remarried (RRR = 1.253; 95% CI: 1.127–1.393). Likewise, the expected risk of intention to use contraceptives later compared with being uncertain was 14.8% among women whose household power relations were male dominated (RRR = 1.148; 95% CI: 1.061–1.243). The selected background characteristics showed varying degrees of association with intention to use contraceptives later. However, while rural residence was associated with higher intention to use contraceptives, advanced reproductive age and Islam were associated with lower intention to use contraceptives later.

## Discussion

This study examined association between grand multiparity and intention to use modern contraceptives in Nigeria. The study advances the frontier of knowledge by addressing the limitation of previous studies which though investigated predictors of intention to use modern contraceptives among women of reproductive age but paid no attention to ascertaining the extent to which grand multiparity is associated with intention to use contraceptives [52–54]. As the country march towards achieving the post-2015 development agenda, it is important not only to recognise that grand multiparity is one of the key reasons that undermine women’s reproductive health through excessive childbearing burden, but also that boosting contraceptive use among them has several health and non-health benefits that could further reposition Nigerian women for more productive role in attaining the post-2015 development agenda in the country. By linking grand multiparity to intention to use contraceptives, this study thus provided an avenue for assessing future contraceptive demand. Though, for some women, intention may not necessarily lead to future use, but for large numbers of women, intention is a sufficient driver of future use.

Understanding the extent of grand multiparity as well as its association with intention to use contraceptives offer additional insights for demographic and health policy makers to develop interventions targeting grand multiparous women in order to improve their reproductive and general well-being. Another merit of the study relates to the quality data analysed. The DHS programmes are well planned and executed across developing countries. This enhances international comparability of findings based on DHS data. Also, the findings in the study provided more support for the TPB in spite of arguments against the TPB [61]. Findings showed that TPB theoretical construct significantly influenced intention to use contraceptives in line with the assertions of the TPB [58–60]. Three of the findings have implications for population and health policy and programming in the country.

One, intention to use contraceptives is low among grand multiparous women in Nigeria. As evident in the study, less than one-fifth of grand multiparous women intend to use contraceptives later. This is not good enough for population and women’s reproductive health in the country for two reasons. Firstly, non-intention to use contraceptives by majority of grand multiparous women implies that they remain vulnerable to unintended pregnancies and high-risk fertility or induced abortion if they have no fertility desire [3, 29], and elevated risk of adverse maternal and child health outcome if they have fertility desire [22–26]. Both possibilities may further overstretch existing poor health delivery system in the country [27–30]. Secondly, additional children by grand multiparous women contributes significantly to current high fertility level in the country and may be one of the reasons slowing down the pace of fertility transition in Nigeria. The transition is assumed to have started in any country when fertility level reduces by at least 10% with an irreversible downward trend. During this period, rise in use of contraceptives is expected among women in higher age groups who are more likely to be grand multiparous in Nigeria. The low intention to use contraceptives among these women as found in the study thus portends serious challenge to fertility transition in Nigeria.

The development of a specific population and health programme to target grand multiparous women is thus imperative in the country. Such a programme could be integrated into existing national family planning programme through specific contraceptive education, counselling and information for high parous women. Also, the programme should seek to ensure that known barriers to contraceptive use in the country such as misconception arising from ignorance or religious practices are fully addressed to enable grand multiparous women with intention to use contraceptives actualise their contraceptive intention. In addition, the programme should evolve strategies built around the factors that improve intention to use contraceptives. As found in this study, the proximate factors that improve intention to use contraceptives are pregnancy termination, fertility planning status, mass media family planning messages, and knowledge of contraceptive, while the underlying factors that interact with parity to improve intention to use contraceptives are remarriage, household power relation, and maternal education.

Two, grand multiparity is dominant among women in Nigeria. This finding corroborates evidence in a number of previous studies in Nigeria which found the dominance of high parity among Nigerian women [37–39, 49]. With increasing evidence that grand multiparity remains an obstetric challenge in Nigeria [40–44], it is important that the consequences of grand multiparity for women’s health be brought to the fore of national population policy strategy for improving women’s health and safe motherhood in the country. Though, the existing programme seeks to devise measures to detect, prevent, and manage high-risk pregnancies and births, however, implementation of the policy has been bedevilled by a plethora of challenges such as very weak enabling environment occasioned by widespread poverty and socio-cultural issues like polygyny, son preference, child marriage, gender-based violence, and child labour, which influences childbearing practices and undermine women’s reproductive health in the country [55, 57]. The on-going review of the policy should be optimally explored by prioritising grand multiparity and its associated consequences.

Three, grand multiparity is negatively associated with intention to use contraceptives in Nigeria. Ordinarily, the higher the number of children ever borne to a woman, the stronger should be her desire to either space or limit childbearing. This will create demand for contraception. But observing that increasing parity reduces intention to use contraceptives as found in the study tends to suggest that while fertility has remained deeply rooted in cultural values and practices, the health and non-health benefits of contraception to women, men and families is yet to be widely appreciated in Nigeria. Though, family planning is voluntary in Nigeria, the connectivity of family planning and higher quality of life for all people needs to be well understood and promoted in all the nooks and crannies of the country not only in ways that deemphasise national focus of national population policy targets, but also in ways that are consistent with the cultural practices of specific majority and minority ethnic groups in the country.

It is important to note that the analyses carried out in the study, as well as inferences drawn from the study may be affected by five factors. One, the study did not predict actual contraceptive behaviour as expected by the TPB, but predicted intention to use contraceptive. The reason for this shortcoming was the use of cross-sectional data in the study which is unable to capture future behaviour. Nevertheless, the study assumed that intention to use contraceptives is an important factor that determines actual contraceptive use in the future. Two, the proximate and underlying factors proxy and analysed in the study may not exactly match the theoretical constructs of the TPB. However, attempts were made in the study to ensure that variables selected as indicators of the TPB constructs are the most appropriate in the available NDHS datasets. Thus, the use of different indicators for the TPB construct in future research may present a different finding. Three, outcome variable in the study was categorical and not ordered as done in some previous studies [60, 62, 63]. This is because data analysed in the study were not derived from bipolar scale as found in some existing studies. However, the use of a different analytical technique compared to techniques in existing studies may have neutralised the effects of differences in the nature of data analysed. Four, the current study focused on women who intend to use contraceptive later. It is also important for future studies to ascertain the factors that drive no intention to use contraceptives so as to devise more contraceptive behaviour change strategies. Five, parity was measured in the study as number of children previously born alive to a woman in line with its description in demographic and population literature [2, 3, 29]. This definition slightly differs from the clinical application of the term by obstetricians and midwives, who considered age of viability of pregnancies as the important feature of parity or nulliparity. Notwithstanding, the definition of parity in the study is relevant because pregnancy and its outcomes is not strictly a clinical matter, but as well a social issue that could be analysed from non-clinical perspective. More importantly, a number of clinical studies have also adopted the definition of parity as number of previously born live births [79, 80]. Hence, the non-clinical definition of parity did not alter the significance of the study findings.

## Conclusion

Findings from the study not only provided evidence that less than one-fifth of surveyed grand multiparous women intend to use contraceptives in the future, it further revealed that maternal grand multiparity is negatively associated with intention to use contraceptives in Nigeria. These findings indicate an unlikely rise in future contraceptive prevalence and significant fertility decline in the country. To boost future contraceptive use, the development of a specific population and health programme to target grand multiparous women is imperative in Nigeria. Such programme could be integrated into existing national family planning programmes through specific contraceptive education, counselling and information for high parous women.
